# Retraction: Hydrogen-rich saline protects myocardium against ischemia/reperfusion injury in rats

**DOI:** 10.3389/ebm.2025.10605

**Published:** 2025-04-10

**Authors:** EBM Editorial Office

Following publication, concerns were raised on the PubPeer platform regarding the integrity of the images in the published figures. Specifically, highlighted sections of the Sham and H2 images in Figure 6 appear to be duplicated.

**FIGURE 6:**
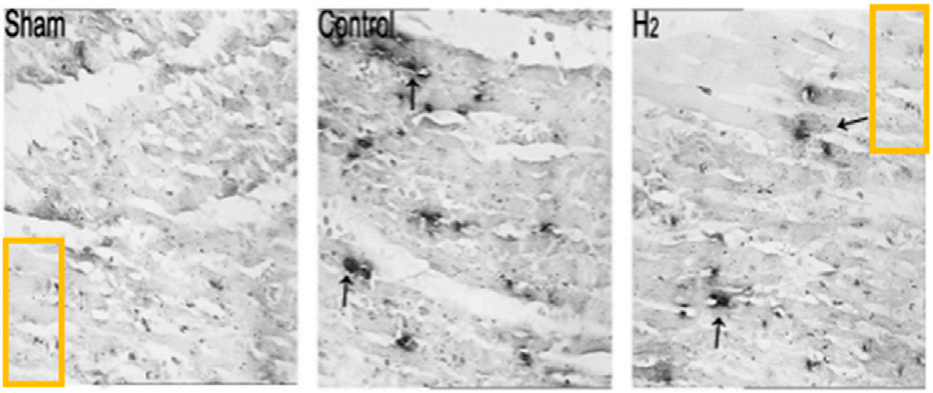
Detection of apoptotic cell death by TUNEL staining in the Sham, Control, and H2 groups at the end of 24 h of reperfusion. Relative to the Control group, H2 significantly reduced the number of TUNEL-positive cells (blue staining). Values are mean ± SEM; P < 0.01 compared to Control group, n = 6 for each group.

The authors remained unresponsive and failed to provide a satisfactory explanation during the investigation, which was conducted in accordance with Experimental Biology and Medicine’s policies. As a result, the data and conclusions of the article have been deemed unreliable, and the article has been retracted.

This retraction was approved by the Editor-in-Chief of Experimental Biology and Medicine. The authors received communication regarding the retraction. EBM would like to thank the users on PubPeer for bringing the published article to our attention.

